# Usefulness of combined use of contrast-enhanced ultrasound and TI-RADS classification for the differentiation of benign from malignant lesions of thyroid nodules

**DOI:** 10.1007/s00330-016-4508-y

**Published:** 2016-08-15

**Authors:** Yan Zhang, Ping Zhou, Shuang-Ming Tian, Yong-Feng Zhao, Jia-Le Li, Lan Li

**Affiliations:** Department of Ultrasound, The Third Xiangya Hospital, Central South University, Changsha, Hunan 410013 China

**Keywords:** Thyroid nodules, TI-RADS, Contrast-enhanced ultrasound, Combination, Improved TI-RADS

## Abstract

**Purpose:**

To study the thyroid image reporting and data system (TI-RADS) classification and the contrast-enhanced ultrasound (CEUS) enhancement pattern of thyroid nodules, and to determine whether combined use of both methods is helpful in the diagnosis of thyroid nodules.

**Methods:**

A total of 319 thyroid nodules in 246 patients were assessed with TI-RADS, CEUS and a combination of both methods. The diagnostic performance of TI-RADS, CEUS and a combination of both methods was compared.

**Results:**

The accuracy in the diagnosis of thyroid nodules was 90.3 % for TI-RADS, 90.0 % for CEUS and 96.0 % for a combination of both methods respectively. A statistically significant difference was not observed in the diagnostic accuracy of CEUS and TI-RADS (*P* > 0.05). However, a significant difference was observed between a combination of both methods and either alone (*P* < 0.01). A combination of both methods showed high sensitivity, specificity and accuracy for TI-RADS classifications of 4a and 4b thyroid nodules compared with TI-RADS alone (*P* < 0.01) and a statistically significant difference was not observed for thyroid nodules classified as 2, 3, and 5 (*P* > 0.05).

**Conclusions:**

The improved TI-RADS, when combined with CEUS, could significantly improve the diagnostic accuracy for thyroid nodules, especially for TI-RADS class-4 thyroid nodules.

***Key Points*:**

• *TI-RADS can be used as the primary diagnostic standard for thyroid nodules*

• *CEUS can be used as an important complement to TI-RADS*

• *The improved TI-RADS can significantly improve the qualitative diagnostic accuracy*

## Introduction

The wide application of ultrasound (US) and other technologies in recent years has led to an increasing number of thyroid nodule diagnoses [1]. The pathological nature of thyroid nodules directly affects the therapeutic methodology and patient prognosis; therefore, the correct diagnosis of thyroid nodules at an early stage has important clinical significance. However, conventional sonographic diagnoses for thyroid nodules presents limitations related to overlapping boundaries, morphologies, internal blood streams, and echoes between malignant and benign nodules. In addition, subjective factors related to the diagnostician can also affect the accuracy of the diagnosis. Therefore, research by Park et al. [2], Horvath et al. [3] and Kwak et al. [4] indicates that the thyroid imaging reporting and data system (TI-RADS), which is based on the breast imaging reporting and data system (BI-RADS), can be used to improve the diagnostic accuracy of thyroid nodules by US, which will provide improvements that can be used in clinical practice.

Contrast-enhanced ultrasound (CEUS) is a focus of current medical US research. CEUS has developed rapidly and is now widely used in many areas of medical US, particularly in diagnoses of liver tumour, and it has greatly improved the specificity and sensitivity of US diagnoses and achieved great clinical value. In recent years, an increasing number of CEUS applications have been developed for thyroid diagnoses in clinical practice, and a number of investigations have been performed to evaluate the different contrast modes in the diagnosis of thyroid nodules. Studies have reported that CEUS can help accurately diagnose both benign and malignant thyroid nodules [5–10].

Currently, studies have reported that both TI-RADS and CEUS can effectively diagnose thyroid nodules; thus, a new scale standard for TI-RADS classification that includes CEUS should be proposed. This paper presents the results of studies performed on 319 thyroid nodules in 246 thyroid patients using TI-RADS, CEUS and combined method with the goal of investigating the value of diagnosing thyroid nodules with the combined CEUS and TI-RADS classification and determining a new and more effective TI-RADS diagnosis standard.

## Materials and methods

### Study subjects

In this study, 246 patients who had received conventional US examinations and CEUS examinations as well as postoperative pathological diagnoses or fine-needle aspiration biopsies (FNABs) between December 2012 and December 2014 in our hospital were recruited. In total, 319 nodule lesions were identified. The patients included 161 women and 85 men; their ages ranged from 19-74 years, and the average was (46.1 ± 15.2) years. The diameter range of the lesions was 2.5-46 mm, and the average was (11.9 ± 3.3) mm. FNAB was performed on 230 nodules, and resection was performed on 89 nodules. The details of the pathological types of thyroid nodules are as follows: 319 nodules included 244 benign nodules and 75 malignant nodules; the benign nodules included 210 nodular goitres, 23 adenomas, 6 adenomatous nodular goitres and 5 inflammatory nodules, and there were 8 thyroid nodules coexisting with Hashimotoʼs thyroiditis in 210 nodular goitres; and the malignant nodules included 58 papillary carcinomas, 8 nodular goitres with complications of local papillary carcinoma, 4 follicular carcinomas, 4 nodular goitres with complications of local follicular cancer and 1 medullary carcinoma. All of the lesions were accompanied by postoperative pathological results or FNAB results. The present study was approved by the hospital ethics committee, and all of the patients provided informed consent.

### Instruments and examination methods

The instrument used in the diagnoses performed here is a Siemens Acuson S2000 colour Doppler US system (Siemens; Mountain View, CA, USA), with contrast pulsed sequencing (CPS) and equipped with an 14 L5 transducer for conventional US and an 9 L4 transducer for CEUS. Every section of the thyroid was carefully scanned, the ultrasonic characteristics of the thyroid structure were observed and recorded, and the images were saved. TI-RADS was used to evaluate and classify every thyroid nodule. The CPS technique and SonoVue contrast agent (Bracco, Milan, Italy) were used. A 20-G needle was inserted into the patientsʼ peripheral veins to establish intravenous access. The contrast agent was mixed with 5 ml saline and shaken to homogeneity, and 2.4 ml of the suspension was then quickly pushed into the peripheral vein with the probe and body position unchanged, and the patient was told not to swallow. The dynamic perfusion process of the lesion was continuously monitored in real time, and the images were saved on the hard drive of the ultrasound diagnosis instrument.

All examinations were performed by an experienced radiologist with more than a decade of experience each in US diagnosis and more than 1 years’ experience of performing CEUS of thyroid nodules in order to exclude the bias from different operators. The US imaging data were respectively analysed by two other experienced radiologists with more than a decade of experience in US diagnosis and with more than 1 years’ experience of performing CEUS. They performed blind independent analyses of the TI-RADS and CEUS images to retrospectively analyse the nature of the thyroid nodules. Then the combined methodology was comprehensively analysed by the third radiologist.

The 4a, 4b thyroid nodules which were categorised by TI-RADS and a combination of TI-RADS and CEUS were studied retrospectively. The correlation between US or CEUS features and pathological findings was analysed statistically. Logistic regression was used to improve the analytical results. The inter-observer variability between experienced and inexperienced examiners using TI-RADS and CEUS was evaluated retrospectively. The US imaging data were independently analysed by an experienced and inexperienced examiner using TI-RADS and CEUS, respectively.

### Diagnostic criteria

#### TI-RADS diagnostic criteria

We have proposed a new classification standard based on the TI-RADS classification criteria proposed by Park et al. [2], Horvath et al. [3] and Kwak et al. [4], and the five ultrasound signs (solid component, marked hypoechogenicity, irregular margins, microcalcifications, taller than wide shape) proposed by Kwak et al. [4]. The new standard was compared with the TI-RADS classification standard proposed by Kwak et al. [4].

Kwak et al. [4] TI-RADS classification criteria:

1. normal thyroid
2. no malignant sign, benign lesions
3. no malignant sign, high probability of benignity
4. one malignant sign; possible benignity
5. two malignant signs; possible malignancy
6. three or four malignant signs; high possibility of malignancy
7. five malignant signs, highly indicative of malignancy

TI-RADS classification criteria in the present study:

1. normal thyroid
2. no malignant sign, benign lesions
3. one malignant sign, high probability of benignity
4. two malignant signs, possible benignity
5. three malignant signs, high probability of malignancy
6. four to five malignant signs, highly suggestive of malignancy

Benign and malignant diagnostic criteria by TI-RADS classification: scores 1-4a are diagnosed as benign and scores 4b-5 are diagnosed as malignant.

#### CEUS diagnostic criteria

In the present study, the CEUS diagnostic criteria are divided into circular enhancement, high enhancement, equal enhancement and low enhancement based on the different US contrast modes. If the contrast indicates high enhancement, circular enhancement or equal enhancement, then the nodule is diagnosed as benign. If the contrast indicates low enhancement, then the nodule is diagnosed as malignant.

#### Improved TI-RADS diagnostic criteria in combination with CEUS

If the CEUS indicates high enhancement or circular enhancement, then one score is subtracted from the TI-RADS classification and score 2 is kept the same. If the CEUS indicates low enhancement, then one score is added to the TI-RADS classification, and score 5 remains the same. If the CEUS indicates equal enhancement, then the TI-RADS classification remains the same.

### Statistical analysis

SPSS 19.0 software was used for the statistical analysis. Quantitative data are expressed as the means ± standard deviation. The χ^2^ test was used to compare the distribution of US indicators for benign and malignant thyroid nodule. The sensitivity, specificity, accuracy, positive prediction value (PPV) and negative prediction value (NPV) of the benign and malignant thyroid nodule diagnoses by TI-RADS, CEUS and combined method were calculated, and the χ^2^ test was used to compare the three methods, with a value of *P* < 0.05 representing significant differences. The diagnostic accuracy of score 2-5 thyroid nodules by a combination of both methods and by TI-RADS alone was compared, with *P* < 0.05 representing significant differences. In addition, logistic regression was used to evaluate the diagnostic performance of the 4a, 4b thyroid nodules, which were categorised by TI-RADS and a combination of TI-RADS and CEUS. Inter-observer variability between experienced and inexperienced examiners using TI-RADS and CEUS were obtained and analysed with the Cohen’s statistic.

## Results

### TI-RADS results

#### TI-RADS diagnostic information

The malignancy rates of TI-RADS score 2, score 3, score 4a score 4b, score 5 were 0,3.0,10.8,67.2, and 92.9 %, respectively (Table 1).

**Table 1:** TI-RADS diagnostic information in the present study

TI-RADS	Number of nodules	Pathological results /number		Malignancy rate %
		Benign	Malignant	
Score 1	0	0	0	0
Score 2	68	68	0	0
Score 3	100	97	3	3.0
Score 4a	65	58	7	10.8
Score 4b	58	19	39	67.2
Score 5	28	2	26	92.9

#### TI-RADS diagnostic classification by Kwak et al.

A comparative study was performed between the classification criteria proposed by Kwak et al. [4] and the classification criteria presented here, and the results show that there is a significant difference between the two classification methods (χ^2^ = 27.9, *P* < 0.01) (Tables 2 and 3).

**Table 2:** Kwak TI-RADS diagnostic information

TI-RADS	Number of nodules	Pathological results/number		Malignancy rate %
		Benign	Malignant	
Score 1	0	0	0	0
Score 2	23	23	0	0
Score 3	45	45	0	0
Score 4a	100	97	3	3.0
Score 4b	65	58	7	10.8
Score 4c	71	19	52	73.2
Score 5	15	2	13	86.7

**Table 3:** Comparison of the diagnostic performance of the two TI-RADS classifications

Examination method	Sensitivity	Specificity	Accuracy	PPV	NPV
Kwak TI-RADS	96.0 %	67.6 %	73.8 %	47.7 %	98.2 %
TI-RADS in the present study	86.7 %	91.4 %	90.3 %	75.6 %	95.7 %

### CEUS results

Among the 75 malignant nodules in the present study, 58 (77.3 %) indicated low-enhancement mode, 13 (17.3 %) indicated equal enhancement mode, 2 (2.7 %) indicated high-enhancement mode and 2 (2.7 %) indicated circular enhancement. Among the 244 benign nodules, 15 (6.1 %) indicated low-enhanced mode, and 229 (93.9 %) indicated equal enhancement, circular enhancement or high-enhancement mode (Table 4).

**Table 4:** CEUS enhancement information on thyroid nodules

CEUS enhancement mode	Number of nodules	Pathological results/number	
		Malignant nodule	Benign nodule
Low enhancement	73	58	15
Equal enhancement	65	13	52
High enhancement	74	2	72
Circular enhancement	107	2	105
Total	319	75	244

### Accuracy of thyroid nodule diagnoses by CEUS combined with TI-RADS

**Fig. 1:**
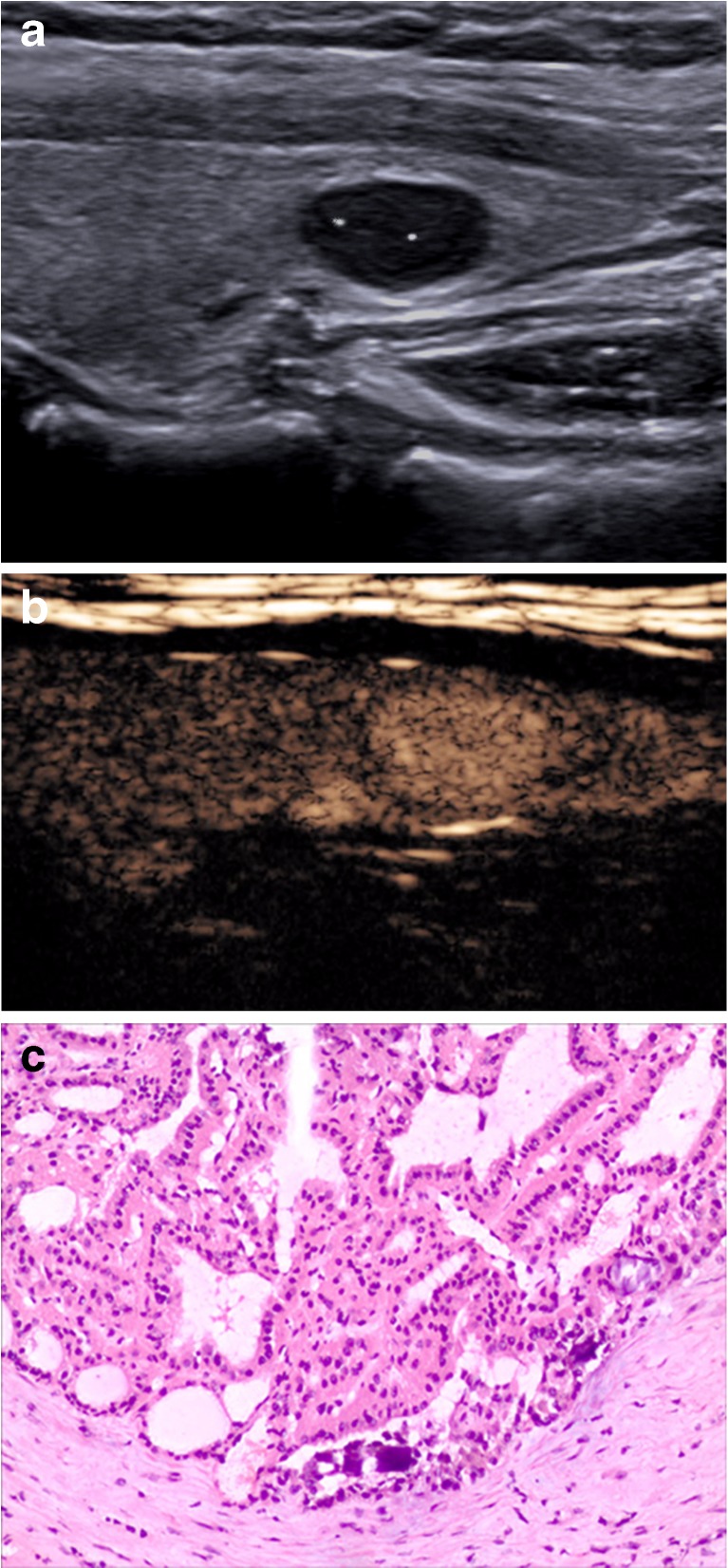
A 35-year-old man was found to have an 8 × 6-mm solid hypoechoic nodule in the left lobe of his thyroid. **a** A conventional two-dimensional image. This nodule had three malignant indicators (solid, markedly hypoechoic, and microcalcifications), and it was classified to a 4b TI-RADS score. **b** The ultrasound contrast status and the enhancement mode indicated high enhancement. The improved TI-RADS combined with CEUS returned a score of 4a, which indicates a benign nodule. **c** The pathological image of the lesion, which was of a nodular goitre

**Fig. 2:**
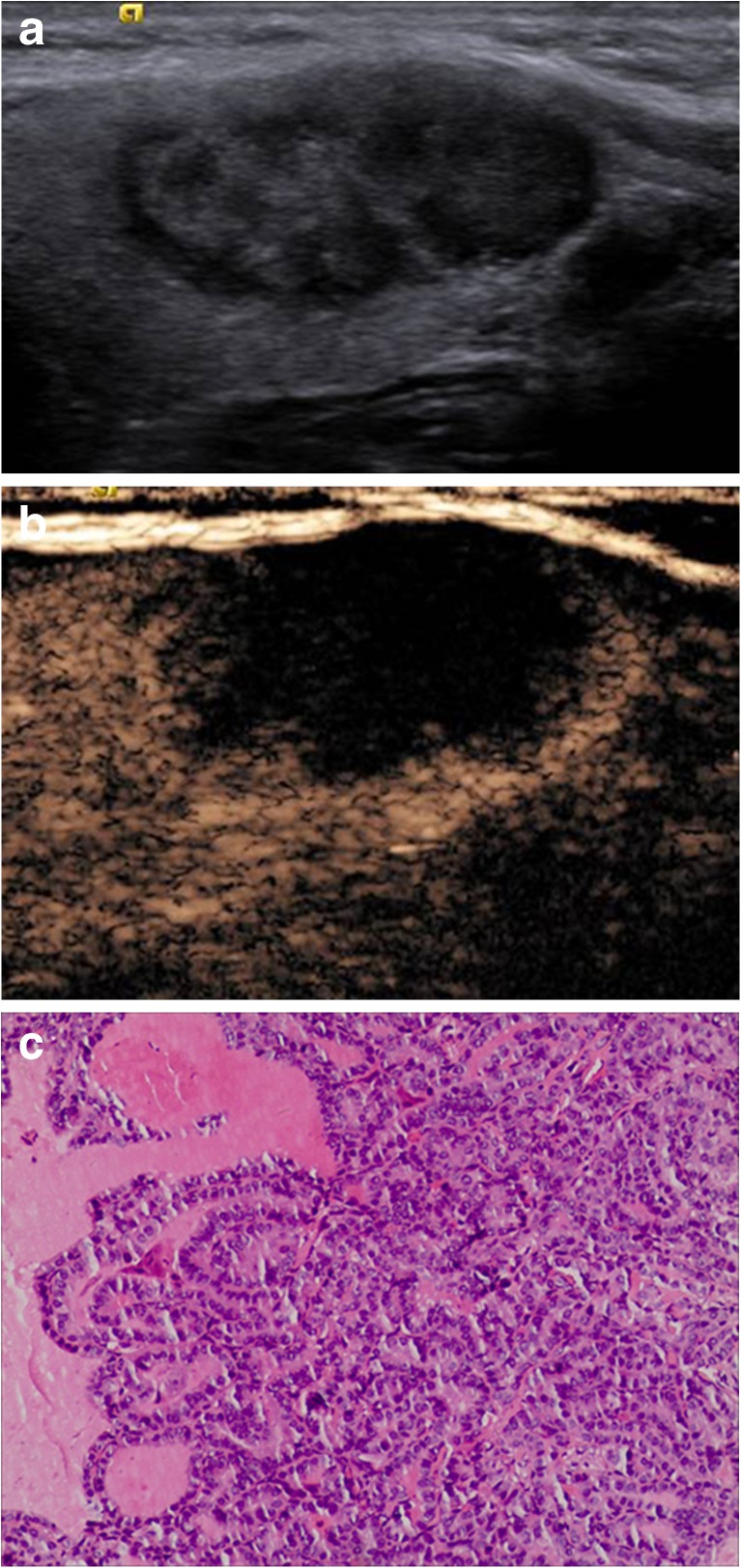
A 42-year-old woman was found to have a 32 × 15-mm solid nodule in the left lobe of her thyroid. **a** A conventional two-dimensional image. This nodule had two malignant indicators (solid and irregular margin), and it was classified to a 4a TI-RADS score. **b** An ultrasound contrast image, and the enhancement mode indicates low enhancement. The improved TI-RADS combined with CEUS returned a score of 4b, and the diagnosis indicated a malignant nodule. **c** The pathological image of the lesion, which is a thyroid papillary carcinoma

**Fig. 3:**
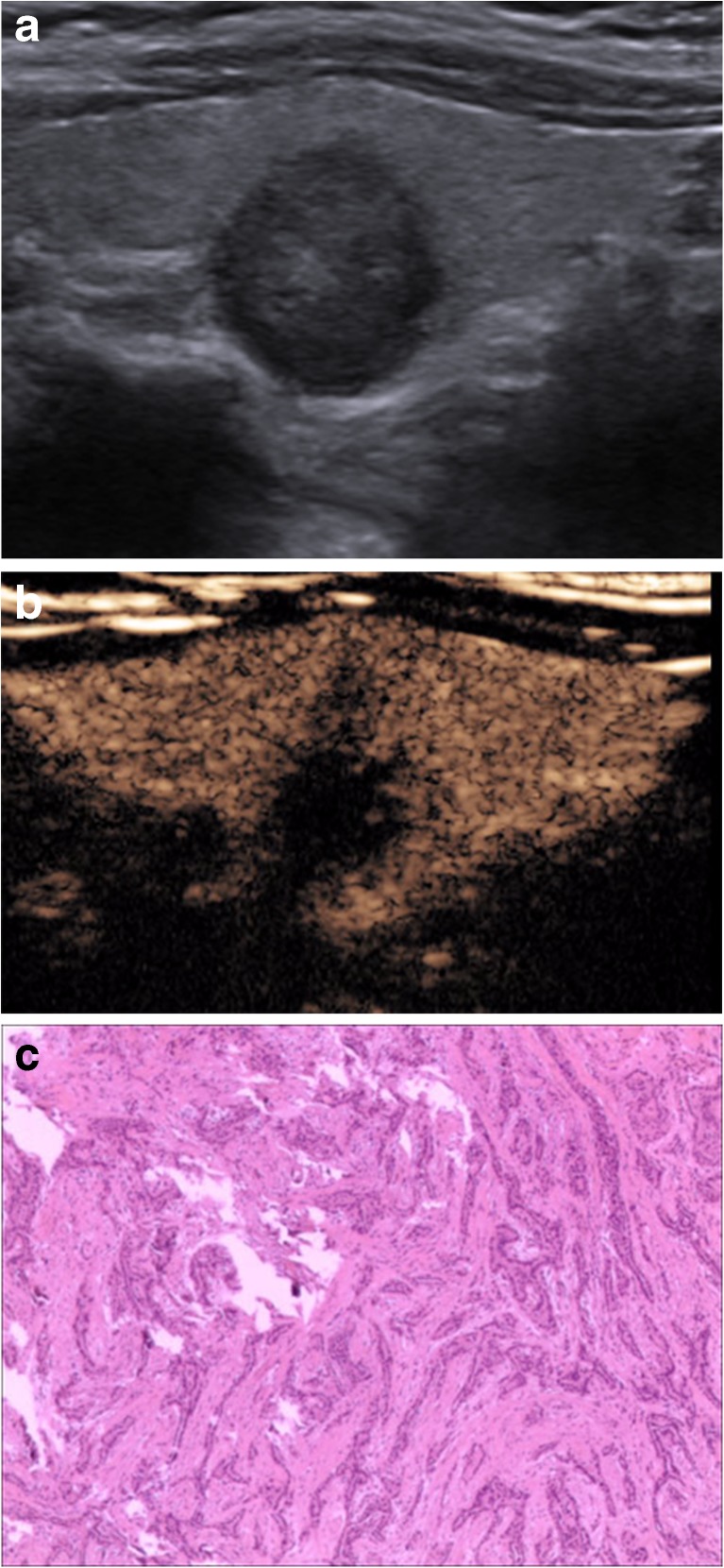
A 38-year-old woman was found to have a 12 × 12.5-mm solid hypoechoic nodule in the left lobe of her thyroid. **a** A conventional two-dimensional image. This nodule had three malignant indicators (solid, aspect ratio greater than 1 and markedly hypoechoic) and was classified to a 4b TI-RADS score. **b** An ultrasound contrast image; the enhancement mode was low enhancement. The improved TI-RADS combined with CEUS returned a score of 5, and the diagnosis indicates a malignant nodule. **c** The pathological image of the lesion, which is a thyroid papillary carcinoma

**Fig. 4:**
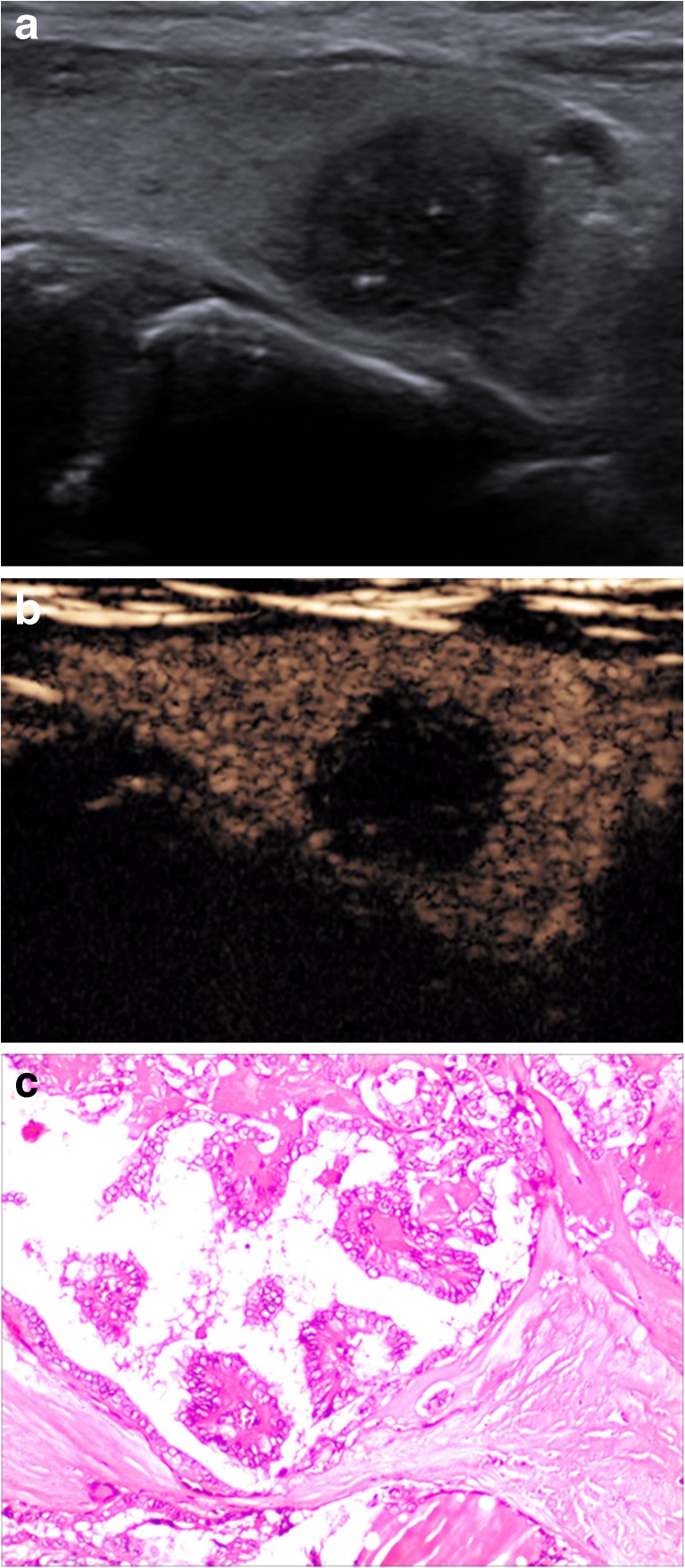
A 48-year-old woman was found to have a 14 × 12-mm solid hypoechoic nodule in the right lobe of her thyroid. **a** A conventional two-dimensional image. This nodule had four malignant indicators (solid, irregular margin, markedly hypoechoic and microcalcifications); it was classified to a TI-RADS score of 5. **b** An ultrasound contrast image; the enhancement mode was low enhancement. The improved TI-RADS combined with CEUS returned a score of 5, and the diagnosis indicates a malignant nodule. **c** The pathological image of the lesion, which is a thyroid papillary carcinoma

When CEUS and TI-RADS were used together for diagnosis, the malignancy rate of nodules classified as 4a and below was decreased compared with that of TI-RADS alone, whereas the malignancy rate of nodules classified as 4b and above was increased compared with that of TI-RADS alone (Table 5). The results from FNAB and pathological tests were used to verify the accuracy of the tested methods, and the sensitivity, specificity, accuracy, PPV and NPV of malignant thyroid nodule diagnoses by TI-RADS, CEUS and the combined method were compared. Significant differences were not observed between the diagnoses by CEUS alone and TI-RADS alone (χ^2^ = 0.01, *P* > 0.05), whereas significant differences were observed between the diagnoses of the combined method and that of TI-RADS alone and CEUS alone (χ^2^ = 9.0, *P* < 0.01; χ^2^ = 9.8, *P* < 0.01) (Table 6, Figs. 1, 2, 3 and 4).

**Table 5:** Diagnoses by CEUS combined with TI-RADS

TI-RADS	Number of nodules	Pathological results/number		Malignancy rate %
		benign	malignant	
Score 1	0	0	0	0
Score 2	78	78	0	0
Score 3	102	101	1	1.0
Score 4a	56	55	1	1.6
Score 4b	52	8	44	75.0
Score 5	31	2	29	90.5

**Table 6:** Diagnostic performance of TI-RADS and CEUS

Examination method	Sensitivity	Specificity	Accuracy	PPV	NPV
TI-RADS	86.7 %	91.4 %	90.3 %	75.6 %	95.7 %
CEUS	77.3 %	93.9 %	90.0 %	79.5 %	93.5 %
TI-RADS+CEUS	97.3 %	95.5 %	96.0 %	88.0 %	99.1 %

### A combination of both methods diagnoses of thyroid nodules classified as 2-5

The results show that a significant difference occurred between the thyroid nodule diagnoses classified as 4a (*P* < 0.01) and 4b (*P* < 0.01) by a combination of both methods and TI-RADs alone, whereas a significant difference was not observed for diagnoses classified as 2, 3 and 5 (*P* > 0.05) (Table 7).

**Table 7:** A combination of both methods of diagnosis of thyroid nodules classified as 2-5

Classification	Method	Effective	Ineffective	*P*
Score 2	TI-RADS	68	0	>0.05
	CEUS+TI-RADS	78	0	
Score 3	TI-RADS	97	3	>0.05
	CEUS+TI-RADS	101	1	
Score 4a	TI-RADS	58	7	˂0.01
	CEUS+TI-RADS	55	1	
Score 4b	TI-RADS	39	19	˂0.01
	CEUS+TI-RADS	44	8	
Score 5	TI-RADS	26	2	>0.05
	CEUS+TI-RADS	29	2	

### Logistic regression analysis

At logistic regression analysis of 4a, 4b thyroid nodules, the following features showed a association with malignancy: solid component, marked hypoechogenicity, irregular margins, microcalcifications, taller than wide shape and low enhancement (Tables 8 and 9).

**Table 8:** Only TRIRADS criteria (4a and 4b lesions)

Parameter	Partial regression coefficient	Standard error	*P* value	Odds ratio
Composition				
Solid	1.519	0.659	0.021	4.566
Mixed				1
Echogenicity				
Marked hypoechogenicity	1.429	0.622	0.021	4.176
Hyper/iso/hypoechogenicity				1
Margins				
Irregular	1.713	0.653	0.009	5.547
Well circumscribed				1
Calcifications				
Microcalcifications	2.337	0.710	0.001	10.353
Macrocalcifications/no calcifications				1
Shape				
Taller than wide	2.542	0.733	0.001	12.710
Wider than tall				1

**Table 9:** TI-RADS with CEUS (4a and 4b lesions)

Parameter	Partial regression coefficient	Standard error	*P* value	Odds ratio
Composition				
Solid	1.961	0.856	0.022	7.106
Mixed				1
Echogenicity				
Marked hypoechogenicity	2.166	0.852	0.011	8.722
Hyper/iso/hypoechogenicity				1
Margins				
Irregular	2.325	0.835	0.005	10.229
Well circumscribed				1
Calcifications				
Microcalcifications	2.487	0.882	0.005	12.029
Macrocalcifications/no calcifications				1
Shape				
Taller than wide	2.652	0.863	0.002	14.182
Wider than tall				1
CEUS				
Hypoenhanced	2.898	0.845	0.001	18.134
Iso/hyper/annular enhanced constant term	-7.743	1.924	0.000	0.000

### Inter-observer variability between experienced and inexperienced examiners

The inter-examiner measurement agreement for TI-RADS and CEUS had a high positive correlation even between experienced and inexperienced examiners (k = 0.885, k = 0.990, respectively, Tables 10 and 11).

**Table 10:** Inter-observer variability of TI-RADS

Observers	2	3	4a	4b	5	k
Experienced examiner	84	97	61	49	28	0.886
Inexperienced examiner	75	107	47	57	33

**Table 11:** Inter-observer variability of CEUS

Observers	Low enhancement	Equal enhancement	High enhancement	Circular enhancement	k
Experienced examiner	75	60	78	106 0.907	0.907
Inexperienced examiner	81	65	72	101	

## Discussion

Early diagnosis, correct identification and diagnosis of the benignity and malignancy of thyroid nodules has important significance for clinical treatment method selections and prognosis predictions. TI-RADS classification identifies the benignity and malignancy of nodules through a comprehensive analysis of nodule morphological characteristics, and this classification provides a standard for the diagnosis of thyroid nodules; however, improvements can be made in its application for the identification of benignity and malignancy among thyroid nodules [2–4, 11]. CEUS is a recently developed ultrasound imaging technology, and studies have shown that monitoring microcirculation perfusions after the injection of microbubble contrast agents can be used to identify the benignity and malignancy of thyroid nodules [5–10]. However, to our knowledge, studies presenting TI-RADS classification criteria that also consider CEUS diagnoses have not been reported. Thus, the primary goal of the present study was to develop a new and effective TI-RADS classification standard by investigating the value of diagnosing thyroid nodules using CEUS combined with TI-RADS and determining whether improvements were made to the diagnostic accuracy.

Currently, a number of scholars have begun applying TI-RADS classifications to thyroid nodules [2–4]; however, a generalised standard has not been developed. In the present study, we have referenced BI-RADS classifications and the classifications by Park et al. [2], Horvath et al. [3] and Kwak et al. [4] to classify thyroid nodules as score 1-5, with score 4 divided into the subscores 4a and 4b. Clinical practice has confirmed that this classification is simple and practical. To develop an objective classification, the American scholar Horvath et al. [3] first proposed a classification using ten US sonographic characteristics, whereas Park et al. [2] proposed using 12 US sonographic characteristics. Recently, Korean scholars Kwak et al. [4] proposed using five ultrasound sonographic characteristics for the classification, and these characteristics are simple and generalised, and highly practical in clinical practice. Therefore, in the present study, we used these five US signs for the classification of each thyroid nodule, although certain flaws were noted [4]. In the classification, malignant indicators are not available for scores 2 and 3; therefore, the difference between these two scores is vague. In the present study, we separated score 2 from score 3 and classified nodules with one malignant sign as score 3. This classification facilitates the standardisation of scores 2 and 3, and reduces the subjectivity of their classification. In addition, the standard presented here classifies nodules with one malignant sign as score 4a and nodules with two malignant signs as score 4b; thus, there may be higher false-positive rates.

To verify the accuracy and effectiveness of the classification standard in the present study, we performed a comparative analysis with the classification method by Kwak et al. [4]. The results showed that our classification method has a higher accuracy than the classification method by Kwak et al. (90.3 % vs 73.8 %). Moreover, because the criteria presented here classify nodules with two malignant signs as score 4a and nodules with three malignant signs as score 4b, the false-positive rate is significantly reduced, which may prevent unnecessary surgical treatment. In the present study, we misdiagnosed 31 nodules based on TI-RADS standards, which accounted for 9.7 % of the diagnoses. The misdiagnosed nodules were mainly in scores 4a and 4b, indicating that these two scores have a degree of overlap.

As an important technique in US medicine, CEUS has played an important role in the diagnosis of thyroid lesions over the last decade [5–10, 12]. Studies from numerous researchers suggest that most of the contrasts indicating low enhancement and heterogeneous enhancement are malignant thyroid nodules [5, 9], whereas most contrast modes indicating high enhancement and circular enhancement are benign nodules [7]. CEUS has been proposed to evaluate the thyroid nodules and the sensitivity, specificity and accuracy for the diagnosis of malignant thyroid nodules were reported to be 76.9–88.2 %, 84.8–95.1 % and 82.6–90.4 % [5–7], respectively. The results presented here are consistent with the results of the above-mentioned studies, which indicates that CEUS has clinical value in the identification and diagnosis of benignity and malignancy of thyroid nodules. In this study, 31 nodules were misdiagnosed based only on TI-RADS standards, and 32 nodules were misdiagnosed based only on CEUS standards. A statistically significant difference was not observed in the diagnostic accuracy of CEUS and TI-RADS. Nineteen misdiagnosed nodules based only on TI-RADS standards were correctly diagnosed by CEUS, and 20 misdiagnosed nodules based only on CEUS standards were correctly diagnosed by TI-RADS. The results indicated that the two methods may have complementary effects in the differential diagnosis of thyroid nodules.

In our study, there were eight thyroid nodules coexisting with Hashimotoʼs thyroiditis in our study, which were all accurately diagnosed by CEUS and partly misdiagnosed by TI-RADS. The result was consistent with that of Zhao et al. [13]. The result indicated that CEUS is also effective in the diagnosis of malignant thyroid nodules coexisting with Hashimotoʼs thyroiditis and can improve the diagnostic accuracy. Of five inflammatory lesions, three nodules showed low-enhancement mode and were misdiagnosed. It indicated that CEUS may not be able to provide more valuable information for inflammatory lesions, and further studies with greater sample size are required to validate the study results.

To increase the accuracy and reduce misdiagnoses as well as missed diagnoses, we have combined CEUS with TI-RADS to identify the benignity and malignancy of thyroid nodules. Significant differences were observed when the combined method was compared with CEUS alone or TI-RADS alone, suggesting that a combined method can significantly increase the accuracy of qualitative diagnoses of thyroid nodules and effectively lower the misdiagnosis rate and missed diagnosis rate. In the present study, we combined CEUS with TI-RADS for further diagnosis analyses and found that nodules with TI-RADS scores of 2, 4 and 5 did not present significant differences (*P* > 0.05) when using the combined diagnosis, whereas nodules with TI-RADS scores of 4a and 4b presented improved diagnoses by a combination of both methods. Among the 56 4a-nodules, 55 were accurately diagnosed, and among the 52 4b-nodules, 44 were accurately diagnosed. These results indicate that a combination of both methods primarily improved the identification and diagnosis of scores 4a and 4b, which are difficult to identify by TI-RADS alone, and it avoids the errors produced by TI-RADS alone. Thus, we suggest that for thyroid nodules, particularly for nodules with a TI-RADS score of 4a and 4b, a combination of both methods should be used for further analysis.

At logistic regression analysis of 4a and 4b thyroid nodules, the following features showed an association with malignancy: solid component, marked hypoechogenicity, irregular margins, microcalcifications, taller than wide shape and low enhancement. These results were consistent with those of Hyobin et al. [14]. It further confirmed that the above features are effective in the diagnosis of malignant thyroid nodules. To use TI-RADS and CEUS on a regular basis in clinical routine it is important that it is highly reliable. The inter-examiner measurement agreement for TI-RADS and CEUS had a high positive correlation even between experienced and inexperienced examiners (k = 0.885, k = 0.990, respectively). It indicated that TI-RADS descriptors and CEUS mode are simple, objective and are less influenced by US physicians’ experience.

Although a combination of both methods presents significant improvements in diagnostic accuracy, 15 nodules were still misdiagnosed, which may have been caused by the following: (1) certain benign nodules may have fibroplasias during hyperplasia and form calcified fibrous septa and calcified nodule walls, thereby affecting the interpretation of CEUS images; (2) the contrast enhancement modes of certain benign and malignant nodules in scores 4a and 4b could have overlapped; (3) two malignant nodules that were diagnosed as benign nodules were the nodular goitres with complications from local papillary carcinoma and from local follicular cancer; this local carcinoma increased the difficulty of providing a correct diagnosis; (4) the pathological results of the two misdiagnosed TI-RADS score-5 nodules were inflammatory lesions, and conventional US and CEUS results for such lesions are similar to malignant nodules, thereby increasing the difficulty of providing a correct diagnosis.

The present study included the following limitations. (1) Most of the malignant nodules were papillary carcinomas, whereas most of the benign nodules were nodular goitres, and fewer additional pathological types were analysed. Therefore, the present study mainly confirms the diagnostic value of TI-RADS and CEUS for papillary carcinoma and nodular goitre, whereas the diagnostic value of these methods for other benign and malignant thyroid pathological types requires further investigation. (2) The present study represents a single thyroid clinic’s work; thus, results have to be confirmed with multi-centre studies and a large sample size.

In summary, CEUS and TI-RADS showed similar accuracy in the identification of benign and malignant thyroid nodules. The improved TI-RADS combined with CEUS can significantly increase the diagnostic accuracy for the qualitative identification of thyroid nodules, particularly for TI-RADS score 4a and 4b lesions, and if these findings can be replicated, then it may be possible to reduce the number of patients subjected to FNAB or short-term follow-up for benign-appearing solid thyroid nodules.

## Abbreviations and acronyms

TI-RADS: thyroid imaging reporting and data system
BI-RADS: breast imaging reporting and data system
CEUS: contrast-enhanced ultrasound
CPS: contrast pulsed sequences
FNAB: fine-needle aspiration biopsy
